# Treatment of Aqueous Effluents from Steel Manufacturing with High Thiocyanate Concentration by Reverse Osmosis

**DOI:** 10.3390/membranes10120437

**Published:** 2020-12-18

**Authors:** José R. Álvarez, F. Enrique Antón, Sonia Álvarez-García, Susana Luque

**Affiliations:** Department of Chemical and Environmental Engineering, University of Oviedo, 33071 Oviedo, Spain; jras@uniovi.es (J.R.Á.); feantonr@gmail.com (F.E.A.); alvarezsonia@uniovi.es (S.Á.-G.)

**Keywords:** reverse osmosis, modeling, thiocyanate, wastewater treatment, process design

## Abstract

The feasibility of reverse osmosis (RO) for treating coking wastewaters from a steel manufacturing plant, rich in ammonium thiocyanate was assessed. DOW FILMTEC^TM^ SW30 membrane performance with synthetic and real thiocyanate-containing solutions was established at the laboratory and (onsite) pilot plant scale. No short-term fouling was observed, and the data followed the known solution-diffusion model and the film theory. Those models, together with non-steady state mass balances, were used in simulations that aided to design a full scale two-stage RO plant for thiocyanate separation.

## 1. Introduction

As part of the steel manufacturing plant, the coke gas generated during the destructive distillation of coal is commonly washed with water at the coke oven exit and is then distributed to several facilities of the steel factory. The condensation of several compounds as the gas temperature decreases requires the use of purge pits placed along the pipe network. This generates an aqueous effluent, designated as coke wastewater in this study, which has to be treated before its definitive disposal. Coke wastewater is expected to contain the same pollutants than a typical refractory industrial wastewater (i.e., those used to quench the coke as it comes out of the blast furnaces or those employed during the cooling and cleaning of the gases), but at different concentrations. These residual streams contain cyanide, thiocyanate, high-strength ammonia, phenolic compounds, heterocyclic nitrogenous compounds and polynuclear aromatics compounds [1,2]. As the biological treatment has become the most reliable option to remove these pollutants, the steel factory has its own wastewater treatment plant in order to provide meaningful control over the detoxification and purification of the coke wastewater. Unfortunately, due to the refractory and inhibitory contaminants present in coking wastewater, the biological treatments are not sufficient.

Coke wastewaters with more than 400 mg/L of thiocyanate cause serious problems in the biological reactor. Phenols and free cyanide seriously inhibit various biological reactions, especially the nitrification reaction [3]. Thus, the biological treatment of the coke wastewater is not as easy as that of domestic wastewaters. To solve these problems, a large amount of NH_4_SCN degradation processes are described in the literature, both chemical [4,5,6] and biological, using the anaerobic and anoxic denitrifiers process previously to the aerobic reactor [1,3,7,8,9]. However, since NH_4_SCN is a salt with several industrial applications: antibiotic fermentations in pharmaceuticals, metal electroplating, flotation agent in metal industries, stabilizer and accelerator in photography, adjuvant in printing, finishing accelerator in fixing baths in textile industries, as a raw material for the production of herbicides and rustproofing compositions, as a water tracer in oil fields [10] and its recovery is presented as a more interesting alternative.

The developed techniques for recovering thiocyanates can be classified in five groups: solvent extraction, distillation, gel filtration, membrane separation and, in recent years, electrochemical treatment methods [11,12,13]. Selective extraction is based on the difference of solubility, using a polar organic solvent that selectively extracts thiocyanate ions, but it requires high energy input for the subsequent recovery of the solvents. Distillation under pressure is a good alternative for obtaining a pure product but also needs a great amount of energy and generates gaseous thiocyanate, which is highly toxic. Gel filtration uses a polymer gel for separation, but it is expensive, slow and inefficient for large volumes. Electrochemical methods such as electro oxidation, electrocoagulation and electroflotation have been reported for the treatment of various wastewaters, and they have various benefits including simple equipment, easy operation, shortened retention time, rapid-settling and decreased amount of precipitate or sludge [14]. Nevertheless, these electrochemical methods are usually applied to the wastewater already biologically pretreated, On the other hand, membrane-based processes can safely separate thiocyanates from a large amount of wastewater with low energy consumption, which makes them suitable for addressing the NH_4_SCN recovery.

Reverse osmosis (RO), forward osmosis (FO) and nanofiltration (NF) have been applied to wastewater from desulfuration of coke oven gas containing NH_4_SCN [11,12], water from the ammonia-N liquor tank of a coke-making plant in India [15] and to an aqueous process stream from acrylic fiber industries containing NaSCN [13,16], respectively. RO has also been used to remove cyanides and ammonium salts, though at concentrations lower than those included in this study [17]. Additionally, when two thiocyanate salts are present, NF and/or RO membranes can be employed not only for recovering them, but also for separating one salt from another [18,19]. Recently, polymer inclusion membranes (PIM) have been also proposed as an appropriate method for cleaning-up of thiocyanate from gold mine waters [20]. Jin et al. (2013) reported a pilot-scale system based in the combined use of a membrane bioreactor as a pretreatment followed by the NF-RO system to treat coking wastewater reduced thiocyanate concentration to a level suitable for industrial reuse [21].

In the present work, several samples of the coke condensates taken from a steel factory were analyzed and found that NH_4_SCN was the most important contaminant.

The experimental part focuses on assessing the performance of a RO commercial membrane with synthetic aqueous solutions of NH_4_SCN and real wastewaters. Based on those experimental results, an appropriate mathematical model of the RO process is to be developed and used in the design of a full-scale RO plant. This RO plant design must treat a condensate flow rate of 50 m^3^/day and produce a permeate with a low thiocyanate content, eligible for the biological wastewater treatment and a highly concentrated NH_4_SCN solution, which can be used as raw material for several applications.

## 2. Materials and Methods

### 2.1. Experimental Equipment and Procedure

There are a variety of RO membranes available in the market, although each type is particularly suited to certain applications. From a preliminary screening (results not shown), a polymeric membrane from DOW FILMTEC^TM^ (Edina, MN, USA) was selected: SW30. A spiral wound module of 2.5″ (diameter) and 40″ length was used for laboratory-scale experiments. Larger spiral modules (4″-40″) were used for the pilot unit tests. Table 1 shows the specifications for both module types. The same modules were used throughout the whole study. No damage was observed, so no replacing was needed.

**Figure 1:**
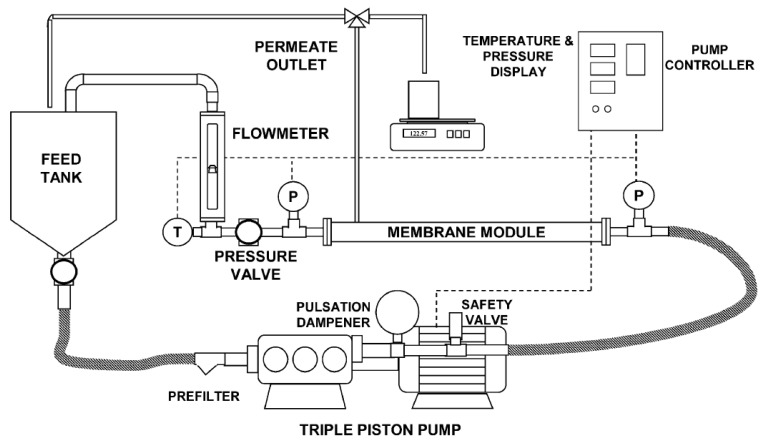
Experimental setup.

Figure 1 shows a diagram of the equipment used to perform laboratory experiments. It consists of a small filtration unit fed from a 100 L tank by a positive-displacement pump. Pressure transducers are placed before and after the module to monitor the pressure in the feed and retentate. Permeate is open to atmosphere. A needle valve is placed after the membrane module to vary the applied pressure. Temperature is monitored using a Pt100 thermoresistance (WIKA, Kingenberg, Germany).

Although the condensates do not contain suspended solids, they do have a large number of suspended tar droplets. Tar is insoluble in most solvents and, thus, it is difficult to clean. It can deposit on equipment and at the inlet of the membrane modules, clogging them. Therefore, basic water filter cartridges (50 and 5 microns) were employed for its removal.

Pressure effect experiments were carried out at the total recycle, that is, retentate and permeate were recycled to the feed tank. Concentration experiments were carried out at constant pressure removing continuously the permeate.

The permeate flux through the membrane (*J*) was calculated using the following equation:

(1) $$J=\frac{M}{A_{m}\cdot \rho \cdot t}$$

where *M* is the permeate mass collected, *ρ* its density, $A_{m}$ the membrane area and *t* the permeation time.

Membrane separation performance was assessed by means of retention (also called rejection), defined as:

(2) $$\mathrm{Retention}\;\left(\%\right)=\left(1-\frac{c_{P}}{c_{F}}\right)\cdot 100$$

where *c_P_* and *c_F_* are the concentrations in the permeate and in the feed, respectively.

The effect of pressure was assessed by conducting an experiment with increasing feed pressure from 10 up to 50 bar, in 10 bar intervals at constant temperature (25, 35 or 45 °C). In the concentration mode experiments, a constant temperature of 25 °C and a pressure of 50 bar were used. Results were analyzed using the solution-diffusion model [22] and the classic film theory [23], with the following mathematical expressions for the overall permeate flux and for the solute flux, respectively:

(3) J=A(ΔP−Δπ)

(4) $$J=k\;\ln\frac{c_{m}-c_{P}}{c_{B}-c_{P}}$$

(5) $$J_{S}=B\;\left(c_{B}-c_{P}\right)$$

where,

*J*: Overall permeate flux (L/m^2^ h).
*J_S_*: Solute flux, *Js=J·c_P_,* (g/m^2^ h).
*k*: Mass transfer coefficient (L/m^2^ h).
*c_m_*: Solute concentration on the membrane surface (g/L).
*c_B_*: Bulk solute concentration in the retentate (g/L).
*c_P_*: Solute concentration in the permeate (g/L).
*A*: Solvent (water) membrane permeability (L/m^2^ h bar).
*B*: Solute membrane permeability (L/m^2^ h).

In the data analysis presented, the solute is the thiocyanate ion. Equation (4) can be simplified when high retention membranes are used in reverse osmosis processes. As concentration in the permeate is generally much lower than that in the retentate, it can be neglected and when *c_m_* is constant, according to the film theory:

(6) $$J=const-k\;\ln\left(c_{B}\right)$$

Water permeability was checked before and after each run by checking tap water flux at five transmembrane pressures (from 10 to 50 bar). The osmotic pressure was negligible and the permeability was calculated as the slope, A, following Equation (3). The difference in water permeability was used as a way to estimate fouling.

Experiments were done also at the industrial site in the vicinity of one of the purge pits, using the same apparatus shown in Figure 1, but equipped with two commercial 4″ RO membrane modules, with an overall filtration area of 15 m^2^ (a six-fold increase from laboratory-scale). The two modules were placed in series, and a water-cooled shell and tube heat exchanger was also located after the modules for improving the temperature control.

Additionally, two 1 m^3^ tanks were used as intermediate storage for the condensate feed to the module and to collect the permeate, respectively. The apparatus was also equipped with two auxiliary centrifugal pumps. One of them was used to pump the condensates out of the purge pit into the first 1 m^3^ feed tank, and the other one was placed between the 100 L tank and the in-line prefilters, before the main high pressure pump, to overcome the prefiltration pressure drop and avoid cavitation at the suction of the high pressure pump.

The water filter cartridges used in the laboratory tests gave poor performance in the industrial installation, so they were replaced by in-line filters, with self-cleaning capability: A Rotorflush RF Series filters, capable of removing suspended solids above 50 microns, allowed maintaining constant flow rates with little maintenance.

Pilot testing lasted one month. It was not run continuously, but in daily 8–10 h shifts, because of the nature of the condensates collection (they were also drawn daily).

### 2.2. Analytical Methods

Samples were analyzed as follows:

- pH and conductivity were determined by a potentiometric method, using a Mettler Toledo Seven Multi Dual pH (Columbus, OH, USA) and conductivity meter.
- Chemical oxygen demand (COD) was determined by refluxing a sample in strongly acid solution with a known excess of potassium dichromate and then measuring the absorbance of the mixture at a wavelength of 620 nm with a HACH DR/2010 spectrophotometer (HACH Co, Loveland, CO, USA) [24].
- Phenolic compounds were characterized by their absorbance at 280 nm [25], with a T80 ultraviolet/visible (UV/VIS) spectrophotometer (PG Instruments Ltd., Leicestershire, UK)
- Total cyanide, free cyanide and weak acid dissociable cyanide concentrations were determined with the same T80 UV/VIS spectrophotometer at 300 nm after the reaction of HCN with chloramine-T, and the addition of a pyridine-barbituric acid agent. The hydrogen cyanide is generated by the alkaline distillation [24] or of ultraviolet radiation [26] on the sample.
- The thiocyanate ion was analyzed by their reaction with Fe(NO_3_)_3_·9H_2_O at low pH, because it forms an intense red color, suitable for colorimetric determination by a PG Instruments Ltd. T80 UV/VIS spectrometer at 460 nm [24].
- The concentration of ammonium was obtained through a potentiometric method, with a selective electrode (Mettler Toledo Type 15 223 3000 Ammonium Electrode) (Columbus, OH, USA) and an Ag/AgCl reference electrode (Mettler Toledo Type 373-90-WTE-ISE-S7) (Columbus, OH, USA) [27].
- SCN^−^ and NH_4_^+^ were also measured through ion-exchange chromatography, together other anions such as SO_4_^2−^, NO_3_^−^ and Cl^−^. A Metrohm Ion Chromatograph 850 Professional IC (Metrohm AG, Herisau, Switzerland) was used, equipped with a Metrosep A Supp 5–100 column for anions, a Metrosep C 3–250/4.0 column for cations and a conductivimeter as a detector.
- Heavy metals and other atomic elements were evaluated by inductively coupled plasma (ICP) in an Agilent 7500 ICP-MS (mass spectrometer) device (Agilet, Santa Clara, CA, USA).

## 3. Results

### 3.1. Analysis of the Samples

Several samples of the condensates were taken from the purge pits during four months, and then, analyzed. Over the first three months, 150 L of the actual condensates were collected weekly, shipped, and used for the lab-scale experiments. Pilot plant experiments were carried out onsite for a month and daily samples were also analyzed. The maxima and minima values of the studied parameters are given in Table 2. The major constituent in the coke wastewaters of this steel company is ammonium thiocyanate. Typical values for thiocyanate ions were between 1 and 3 g/L. Cyanide concentrations have not been included because they were always under the detection limit, 1 mg/L.

**Figure 2:**
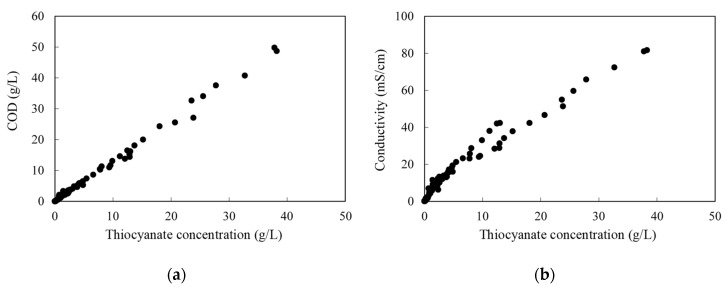
Chemical oxygen demand (COD; (**a**)) and conductivity at 25 °C (**b**) as a function of the thiocyanate concentration.

Both thiocyanate and phenols can be oxidized, thus contributing to the COD load, but after an examination of the compiled data, it was concluded that the phenolic effect was rather limited, being the most of the COD associated with the thiocyanate concentration (an almost linear relation between thiocyanate content and COD can be seen in Figure 2a). During these experiments, it was also observed that the conductivity was mainly influenced by the thiocyanate concentration (Figure 2b). This was useful for designing the experimental work, as conductivity is an easy parameter to follow.

Ammonium thiocyanate is highly soluble in water (over 1.5 kg/L), and at high concentrations (above 300 g/L) conductivity does not behave proportionally to concentration (this was experimentally checked). This behavior was also found in concentrated sodium thiocyanate solutions [28]. However, the latter exhibits a maximum in conductivity that was not found in this work for the ammonium salt.

### 3.2. Effect of Operating Variables with Synthetic Solutions

**Figure 3:**
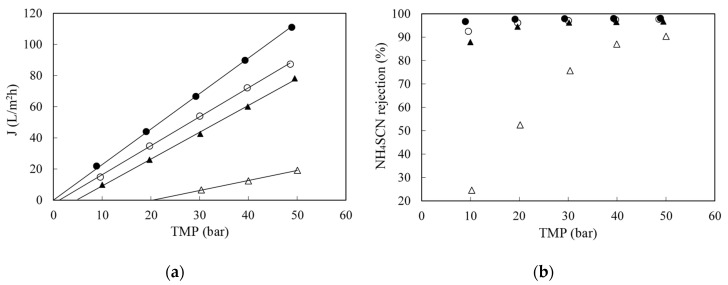
Effect of concentration on permeate flux (**a**) and on NH4SCN retention (**b**) when filtering synthetic solutions of ammonium thiocyanate at 25 °C with the SW30-2540 RO membrane. (●): 1 g/L; (○): 5 g/L; (▲): 10 g/L and (△): 50 g/L.

The effect of pressure on RO of synthetic NH_4_SCN solutions between 1 and 50 g/L in the temperature range 25–45 °C was evaluated in the total recycle mode. Figure 3a shows the effect of pressure at 25 °C. As the concentration increased, the permeate flow decreased, since polarization and osmotic pressure increased. The former resulted in a lower slope, while the latter implies that more pressure had to be applied in order obtain flux through the membrane. For a given concentration, as the transmembrane pressure increased, the permeate flux also increased. No membrane fouling was seen, as water permeability before and after each experiment was the same.

As concentration increased, salt rejection also decreased, as it can be seen in Figure 3b. This is the result of a decreased water flux, as shown in Figure 3a together with an increase in solute diffusion, owing to the larger concentration difference across the membrane (the latter is the driving force for diffusional transport). Therefore, the thiocyanate concentration plays a very important role in the filtration process. As it increases, it actually influences negatively the performance of the process, decreasing the filtration rate and salt rejection.

The experimental osmotic pressures computed from the linear regression of the data shown in Figure 3 were compared with those calculated by the Morse (a modified Van’t Hoff) equation [29]. assuming that NH_4_SCN was fully dissociated. The Morse equation gave values higher than the experimentally determined ones at any condition. That is an indication that the salt is not entirely dissociated.

**Figure 4:**
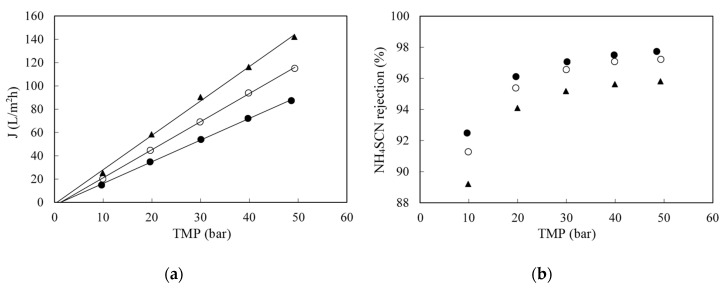
Effect of temperature on permeate flux (**a**) and on NH_4_SCN retention (**b**) when filtering a 5 g/L NH_4_SCN synthetic solutions with the SW30-2540 RO membrane. (●): 25 °C; (○): 35 °C and (▲): 45 °C.

The effect of the temperature on permeate flux can be seen in Figure 4a. Within the concentration range studied, the higher the temperature, the higher the permeate flux. It can be explained by the reduced viscosity, which reduced the resistance of water transport through the membrane and higher membrane permeability. However, as temperature increased, ammonium thiocyanate retention decreased (Figure 4b). This is again inherent to the nature of the separation process, in which the solute transport through the membrane is a function of the solute solubility and diffusion coefficient in the membrane. Both solubility and diffusivity increased exponentially with temperature, so the solute permeability (the product of solubility and diffusivity) increased to a much larger extent than the water flux (associated with viscosity). The result was the observed reduced retention.

### 3.3. Effect of the Transmembrane Pressure with Real Wastewaters

**Figure 5:**
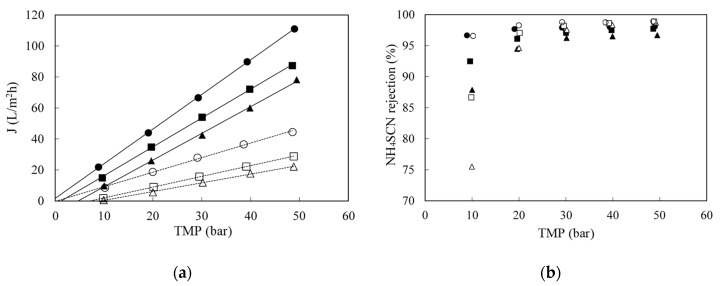
Effect of concentration on permeate flux (**a**) and on NH4SCN retention (**b**) when synthetic solutions (S) and real coke gas condensates (R) are filtered at 25 °C with the SW30-2540 RO membrane. (●): S-1 g/L; (○): R-0.8 g/L; (■): S-5 g/L; (□): R-4 g/L; (▲): S-10.0 g/L and (△): R-9.0 g/L.

The fluxes obtained with the real coking wastewaters were lower (Figure 5a) than those obtained with synthetic solutions, but they followed the same trends with respect to pressure and feed concentration. However, rejections were similar at and above 20 bar, as shown in Figure 5b. A minimum pressure of 20 bar is required for ensuring rejections above 90%. The results shown are an extract of those performed with the weekly samples.

**Figure 6:**
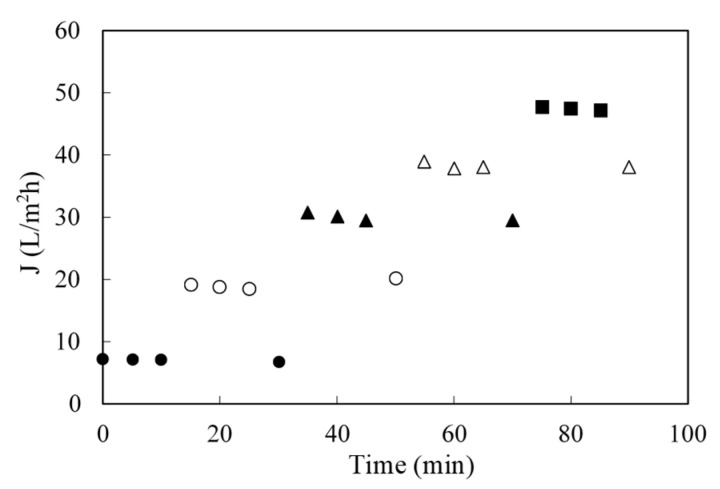
Effect of transmembrane pressure (TMP) on flux stability at 25 °C. Membrane SW30-2540 and NH_4_SCN concentration around 1 g/L. TMP were (●): 10 bar; (○): 20 bar; (▲): 30 bar; (△): 40 bar; and (■): 50 bar.

To account for fouling, water permeability was measured before and after each filtration. No significant change was observed and no chemical cleaning was needed. The same RO module was used for all experiments. To further analyze fouling, a hysteresis experiment was also carried out, following the evolution of flux with time at each transmembrane pressure (TMP) was monitored as shown in Figure 6. An operating pressure was selected and the permeate flux was measured for over a period of time (normally flux declines with time, so data was collected until stable flux was reached); then, the pressure was increased to achieve a new stable permeate flux and, before proceeding to the higher next pressure it was decreased to its previous value to account for irreversible fouling owing to increased pressure [30].

Since the flux remained stable and there was no decrease in water permeability, it can be concluded that there was no significant (short-term) fouling observed. Flux decline with respect to water flux was, thus, associated with the reversible phenomena, i.e., polarization. The ammonia content and high pH of the condensates, together with a lack of organic matter and scaling inorganic salts were believed to be the main reason for flux stability. This very positive result marked the industrial viability of the RO treatment, since membrane fouling (and the subsequent flux decayed with time) is a key challenge and obstacle for applying membrane technologies to industrial processes [31]. However, for a full-scale plant it is recommended to install—as backup—a clean-in-place unit, for eventual rinsing, cleaning or maintenance of the RO plant.

### 3.4. Effect of Feed Concentration with Real Wastewaters

**Figure 7:**
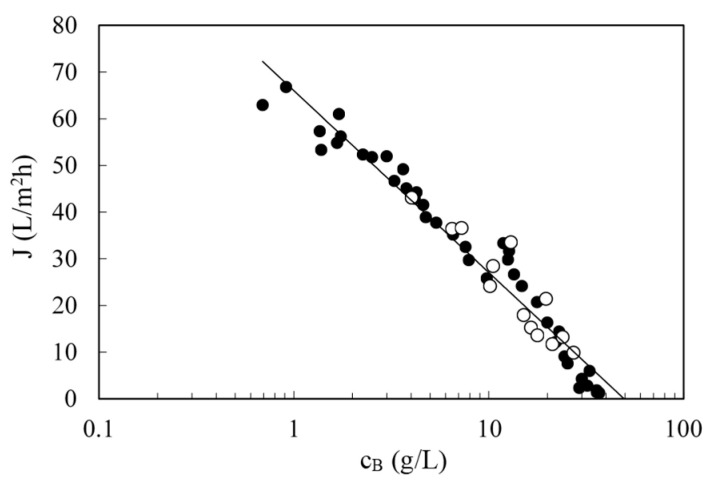
Permeate flux as a function of retentate concentration for several runs. Data collected at 50 bar and 25 °C. Filled circles are data obtained in the laboratory and empty circles are data obtained in onsite pilot tests at the steel factory. Solid line is the fitting to Equation (6).

Due to the variations in the initial concentration (in the weekly samples and in the daily condensates fed to the pilot unit), fluxes also differed from day to day at the same pressure. When all data were plotted together, flux decreased with concentration (Figure 7). Laboratory (black circles) and pilot scale (open circles) results followed the same trend. Flux follows a logarithmic dependence with concentration, as film theory predicts (Equation (4)). The constant term in Equation (6) and the mass transfer coefficient can be calculated from the fitting line, being 66.0 L/m^2^ h and 16.9 L/m^2^ h, respectively.

**Figure 8:**
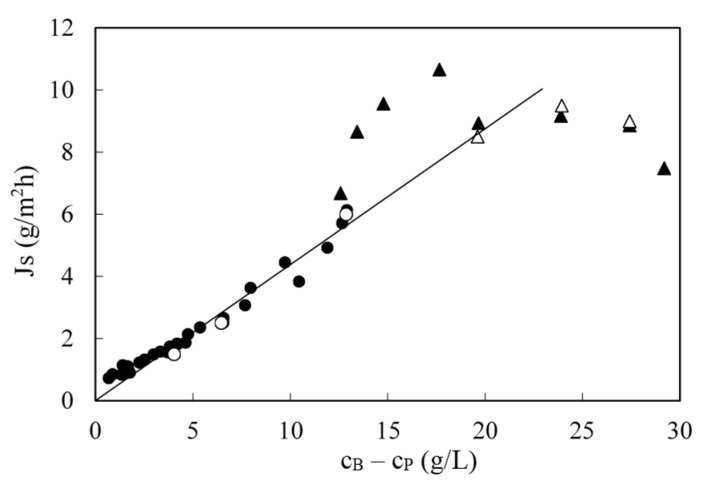
Solute flux through the membrane as a function of the difference between retentate and permeate concentrations for several runs. Data collected at 50 bar and 25 °C (circles) and at various temperatures (triangles). Filled and empty symbols have the same meaning that in Figure 7. Solid line is the fitting of circles to Equation (4).

To calculate the solute permeability, a plot of the solute flux vs. the concentration difference between the retentate and permeate is required as shown in Figure 8. This figure, again, includes data from multiple runs. The slope of the straight line was *B* (0.438 L/m^2^ h) according to Equation (5) of the solution-diffusion model. At higher concentrations, towards the end of the batch concentration runs, implying lower volumes in the feed tank, temperature was more difficult to control and there was more data scattering (shown as triangles). Those values were not included in the regression.

The initial assumption that permeate concentration was negligible seems reasonable only at low feed concentrations. When retentate concentration rose, solute flux through the membrane also increased leading to a rise in permeate concentration, which could reach non-negligible values. Therefore, for the complete validation of the model, a batch concentration operation was simulated using non-steady mass balances and the transport equations, and compared with experimental data. Firstly, it was taken into account that only the permeate stream left the process. Thus, the overall and solute non-steady mass balances can be written as follows:

(7) $$\frac{dV}{dt}=-J\;A_{m}$$

(8) $$\frac{d\left(V\cdot c\right)}{dt}=-J\;A_{m}\;c_{P}$$

where *V* is the volume of the feed/retentate remaining in the tank at a given time, *c* the thiocyanate concentration in the feed tank at the same given time and $A_{m}$ the membrane surface area. Developing Equation (8) and substituting Equation (7) in it results in the following expression:

(9) $$\frac{dc}{dV}=-\frac{\left(c-c_{P}\right)}{V}$$

Combining Equations (5) and (6), and considering that the solute and solvent flux are related by the solute concentration in the permeate, *Js = J.c_P_*, the resulting expression is as follows:

(10) $$c_{Pi}=\frac{B\;c_{i}}{B+const-k\ln\left(c_{i}\right)}$$

**Figure 9:**
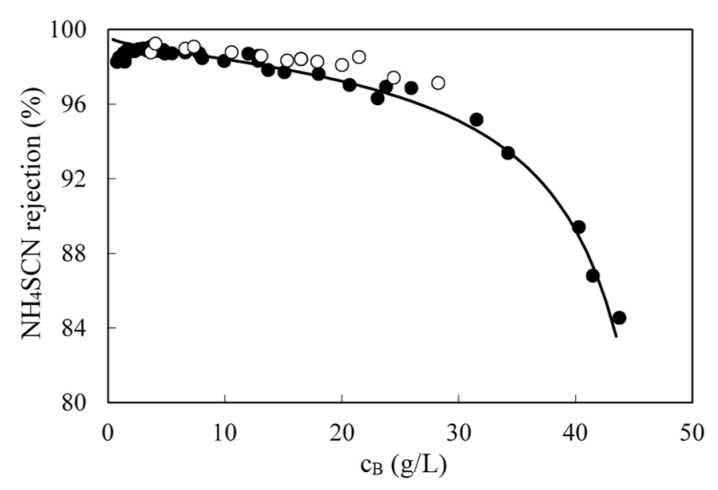
Influence of SCN-concentration on the membrane rejection of this ion. Filled circles are data obtained in the laboratory, empty symbols are experiments done in the steel factory and the solid line is the model prediction.

The numerical integration of Equation (9) along with Equation (10) with the experimentally determined values of *B*, *const* and *k*, allowed the mathematical description of the complete RO process. As it can be seen in Figure 9, the proposed transport model fit successfully the experimental results over the whole concentration range. Therefore, this model will be used for the design of the full-scale RO plant.

### 3.5. Design of a RO Plant for Continuous Operation

Based on the experimental results and the model, a full scale RO plant was designed. The plant must treat a condensate flow rate of 50 m^3^/day and produce a permeate with a low thiocyanate content, eligible for the biological wastewater treatment and a retentate concentrated in NH_4_SCN. The RO plant design began with the easiest assembly, a single stage. The operation mode chosen for the system was fed and bled (a part of the retentate stream is recycled to the stage input) since it allows working in the best hydrodynamics conditions. Based on the aforementioned limited capability of the biological digestion to treat thiocyanate, the target maximum permeate concentration was set at 300 mg/L (0.3 g/L).

Initial values needed for solving the corresponding model equations were the feed flow rate and feed concentration. The feed flow rate was set at 50 m^3^/day. As thiocyanate concentrations falls typically in the range 1–3 g/L, but at times the concentration may spike to 9 g/L, those three values were chosen for calculating different case studies. The membrane area and water recovery (ratio between permeate flow and feed flow) were varied and the resulting permeate concentrations calculated. In order to achieve high recovery, upstream concentration has to increase. One stage was unable to meet the maximum allowed permeate concentration when the concentration was above 10 g/L.

To overcome this restriction, which is inherent to the lack of flexibility of a single stage design, a two-stage RO process with internal recycle was then proposed. The first stage treats the condensates, and produces a permeate stream, which always meets the concentration restrictions for reuse or biological treatment. The second stage allows the concentration of thiocyanate in the retentate stream to a greater extent, while yielding a permeate stream, exceeding the 0.3 g/L, that is internally recycled to the front end of the RO process.

**Figure 10:**
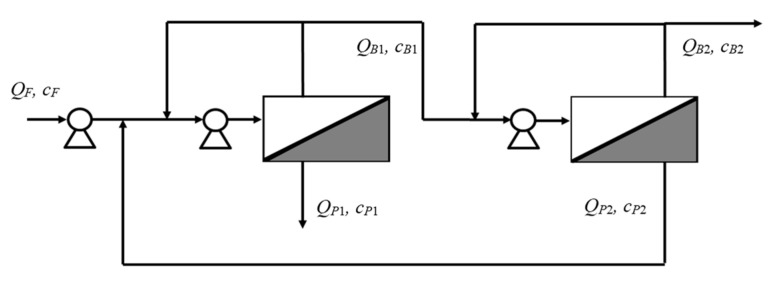
The two-stage reverse osmosis process working in the feed and bleed mode with internal recycle.

The operating mode of each stage continues to be fed and bled. Thus, the retentate stream of each stage is recycled back at the beginning of each stage respectively to allow proper hydrodynamics conditions as shown in Figure 10.

The design equations used, in addition to the solution-diffusion and film theory expressions given in Equations (4) and (10), are the overall and solute mass balances for the two stage process:

(11) $$Q_{F}=Q_{B2}+Q_{P1}$$

(12) $$Q_{F}\;c_{F}=Q_{B2}\;c_{B2}+Q_{P1}\;c_{P1},$$

and the balances around the recycled permeate stream and first stage:

(13) $$Q_{B1}+Q_{P1}=Q_{F}+Q_{P2}$$

(14) $$Q_{B1}\;c_{B1}+Q_{P1}c_{P1}=Q_{F}\;c_{F}+Q_{P2}\;c_{P2}$$

where *Q* denotes flowrate, *c* denotes concentration at the stream locations indicated in Figure 10.

The required membrane area, $A_{m}$, for each stage *j*, can be calculated from the ratio of permeate flowrate and permeate flux:

(15) $$A_{mj}=\frac{Q_{Pj}}{J_{j}}$$

In order to solve the above set of equations, it was necessary to define the feed flow rate, feed concentration (1, 3 or 9 g/L) and two more parameters, one for each stage, respectively. For the first one, permeate concentration was specified (0.3 g/L). For the second one, permeate concentration was also selected and matched to be the same as the fresh condensate feed entering the first stage, although other options are possible for this second variable (i.e., a given thiocyanate concentration in the final retentate to facilitate further processing). The optimization criterion was to minimize the total membrane area (as the sum of the areas of stage 1 and 2, $A_{m1}$ and $A_{m2}$).

After the corresponding calculations, it was found that $A_{m1}$ and $A_{m2}$ were 50 and 41 m^2^, respectively. For this design, Table 3 indicates volumetric flowrates and concentrations for different feed concentrations. The designed plant would be able to treat condensates in within the studied (1–9 g/L) thyocianate feed concentrations always meeting the design constrains by adjusting the recovery. In practice, this can be easily automated with an in-line conductivity sensor.

## 4. Conclusions

Reverse osmosis using commercial SW30-2540 membrane, from DOW FILMTEC^TM^, was proved to be a feasible technology in the recovery of thiocyanates from the coke gas condensates. The transmembrane pressure, temperature and feed concentration were the main operating variables. The permeate flux increased with temperature and transmembrane pressure but decreased with the feed concentration. On the other hand, NH_4_SCN rejection increased with pressure (especially at low feed concentrations), but decreased with concentration and temperature.

The pilot plant runs were conducted to validate the lab-scale experiments with fresh condensate feed, which typically show daily variation in concentration, and was a major concern for plant performance. Confirmation of the absence of short-term fouling observed in lab-scale experiments was also targeted. No fouling was observed throughout all the pilot runs carried out, despite the variable feed concentration and temperature. Therefore, no cleaning cycles were needed. However, for a full-scale plant it is recommended to install—as backup—a clean-in-place unit, for eventual rinsing, cleaning or maintenance of the RO plant. The precaution that was required in the onsite testing was a prefiltration step to remove suspended tar particles.

Experiments performed with samples in the laboratory and with fresh wastewater in the steel factory were in good agreement. The solution-diffusion model and the classic film theory can be combined for representing the process, fitting only three parameters. These models together with mass balances were applied for designing a full RO plant for treating 50 m^3^/day of condensates in a continuous mode (with NH_4_SCN concentrations in the range 1–3 g/L, but that can reach eventually values of 9 g/L). The most appropriate design has two stages with internal recycle, with 50 m^2^ of membrane area in the first stage and 41 m^2^ in the second stage. In this way, permeates with concentrations of thiocyanate under 0.3 mg/L and retentate streams concentrated up to more than 20 g/L were obtained.

## Figures and Tables

**Table 1:** Membrane specifications (according to the manufacturer).

Membrane Module	Nominal Active Surface Area (m^2^)	Maximum Feed Flow Rate (m^3^/h)	Stabilized Salt Rejection ^1^ (%)
SW30-2540	2.8	1.4	99.4
SW30-4040	7.4	3.6	99.4

^1^ Measured at 32,000 mg/L NaCl, 55 bar, 25 °C and 8% recovery.

**Table 2:** Analyses of condensates collected during the sampling period.

Parameter/Element	Minimum	Maximum	Element	Minimum	Maximum
pH	8.6	9.1	Ca (mg/L)	0.6	3.5
Conductivity (mS/cm)	4.8	14.6	K (mg/L)	0.6	2.7
COD (g/L)	0.6	3.2	Si (mg/L)	0.0	2.1
SCN^−^ (g/L)	0.2	9.0	Br (mg/L)	0.1	0.6
NH_4_^+^ (g/L)	1.0	12.9	I (mg/L)	0.0	0.2
Phenols (measured as absorbance at 280 nm)	1.16	3.55	Sr (mg/L)	0.0	0.1
			Al (mg/L)	0.0	0.2
SO_4_^2−^ (mg/L)	130	1007	Cu (mg/L)	0.0	5.0
Cl^−^ (mg/L)	6	50	Zn (mg/L)	0.0	0.1
NO_3_^−^ (mg/L)	12	60	As (mg/L)	0.0	0.1
Fe (mg/L)	12	30	B (mg/L)	0.0	0.2
Mg (mg/L)	0.9	13.6	Ba (mg/L)	0.0	0.5
Na (mg/L)	1.3	6.6	Ge (mg/L)	0.0	0.3

**Table 3:** Volumetric flowrate in L/h (thiocyanate concentration in g/L) for the selected two-stage reverse osmosis process design.

*Q_F_* (*c_F_*)	*Q*_*P*1_ (*c*_*P*1_)	*Q*_*B*1_ (*c*_*B*1_)	*Q*_*P*2_ (*c*_*P*2_)	*Q*_*B*2_ (*c*_*B*2_)
2083 (1.0)	2007 (0.050)	525 (4.6)	449 (1.0)	76 (26.0)
2083 (3.0)	1750 (0.078)	1025 (6.3)	691 (0.5)	334 (18.0)
2083 (9.0)	1176 (0.226)	1525 (12.4)	617 (0.6)	907 (20.4)
